# TikTok in Plastic Surgery: A Systematic Review of Its Uses

**DOI:** 10.1093/asjof/ojad081

**Published:** 2023-09-01

**Authors:** Alexander Zargaran, Sara Sousi, David Zargaran, Afshin Mosahebi

## Abstract

TikTok (San Jose, CA) is a popular and rapidly growing social media platform. With beauty and skincare among the top 5 most popular categories, TikTok represents an important platform for plastic surgery education and communication. However, given the vast array of content shared daily, regulating content for veracity is challenging. It may also be an important and potentially overlooked avenue for the dissemination of inaccurate information pertaining to plastic surgery. This systematic review evaluates TikTok's impact on plastic surgery. Following Preferred Reporting Items for Systematic Reviews and Meta-Analysis Guidelines, a systematic literature review was performed of the use of TikTok within the plastic surgery field. The following databases were queried: PubMed (National Institutes of Health; Bethesda, MD), EMBASE (Elsevier; Amsterdam, the Netherlands), and PsychInfo (American Psychological Association; Washington, DC). The search captured 31 studies of which 7 were included in the final analysis. The studies examined the following areas: gender-affirming surgery (*n* = 1), breast reconstruction (*n* = 1), aesthetic surgical procedures (*n* = 1), plastic surgeon profiles (*n* = 1), and profiles of videos relating to plastic surgery hashtags (*n* = 3). The videos’ quality was assessed using the DISCERN scale. Physician videos scored notably higher than nonphysician videos. The mean DISCERN score across all the videos (*n* = 386) was 1.91 (range: 1.44-3.00), indicating poor quality. TikTok is a popular medium for sharing plastic surgery content. The existing literature has demonstrated overall poor-quality information on plastic surgery, and further study is needed to evaluate its impact in terms of perceptions of the specialty and healthcare behaviors. Future work should focus on promoting accurate, high-quality videos, potentially including a peer-review function for healthcare content. This can leverage TikTok's potential for disseminating content while upholding patient safety.

**Level of Evidence: 3:**

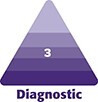

Social media platforms have seen an increase in healthcare-related content, with TikTok (San Jose, CA) currently the second most used platform after Instagram at 29%, with over 1.6 billion monthly active users.^1,2^ Specifically, beauty and skincare account for 1 of the top 5 most popular categories,^3^ thus TikTok represents an important platform for plastic surgery education and communication. A feature that amplifies TikTok's reach as a social media platform is that one does not need an account, nor to follow the content creator, to view the videos posted unless those are private.

TikTok is also routinely used and classified as an advertising and revenue-generating platform; in 2022, it generated $11 billion in advertising revenue that equated to a 200% increase from 2021.^4^ It is also the most common channel used by most brands for influencer marketing, and it is expected to deliver the best return on investment based on short-video production.^5^

Furthermore, prospective patients might turn to social media for their research and potential identification of a plastic surgeon or cosmetic influencer. A survey conducted by Landeen et al found that 80% of facial plastic surgeons, who were members of the American Academy of Facial Plastic and Reconstructive Surgeons (AAFPRS), utilize social media in a professional manner.^6^ However, given the vast amount of content created and disseminated on a daily basis, regulating content for veracity is challenging. Therefore, TikTok may also be an avenue for the dissemination of false information pertaining to plastic surgery, with important patient safety ramifications. In view of its growing influence in the field, this systematic literature review evaluates the current impact of TikTok on plastic surgery.

## METHODOLOGY

A systematic literature review was carried out to identify any studies exploring TikTok's content within the plastic surgery field. Title and abstract screening, full-text review, and data extraction were carried out independently by two authors using COVIDENCE review management software (Melbourne, Australia).^7^ Any conflict was resolved by a third independent reviewer. The kappa score for interobserver reliability was 1. The Preferred Reporting Items for Systematic Reviews and Meta-Analysis protocol^8^ was followed. This review was registered on PROSPERO^9^ (registration number: CRD42023397054).

### Search Strategy

The search strategy was applied to PubMed/MEDLINE (National Institutes of Health; Bethesda, MD), EMBASE (Elsevier; Amsterdam, the Netherlands), and PsychInfo (American Psychological Association; Washington, DC) from inception until 2022, to identify eligible studies. The search strategy included a combination of the following terms: TikTok, plastic surgery, craniofacial surgery, burns, breast surgery, microsurgery, aesthetic surgery, cosmetic surgery. Any studies that were not identified by the search but known to us as of interest were included. The latest search was conducted on January 15, 2023.

### Study Selection

Studies in English in the field of plastic surgery on the social media platform TikTok were included. Nonprimary research, abstracts, and those not relating to the field of plastic surgery and/or the social media platform TikTok were excluded.

### Data Extraction

The data extraction, collection, and management were carried out independently by two authors using a data extraction form via COVIDENCE. Any disagreement was resolved by a third independent reviewer. The following data were extracted: study characteristics (authors, year of publication, country of origin, and journal), number of videos and their statistics (likes, comments, and shares), domain of plastic surgery, content type, and assessment of the quality of videos (DISCERN score). The DISCERN instrument compromises of 16 questions divided into three sections, namely reliability, quality, and overall score. The DISCERN score scale is from 1 to 5, where a higher score equates to higher quality information; the classification is defined as follows: excellent (4.2-5), good (3.4-4.1), average (2.6-3.3), poor (1.9-2.5), and very poor (<1.8).

### Quality Assessment

Risk of bias assessment was performed by 2 reviewers independently using the Crowe Critical Appraisal Tool (CCAT).^10^ Where there were discrepancies, they were resolved by a third reviewer. The CCAT is split into 8 categories and each one receives a score on a 6-point scale from 0-5, with 0 being the lowest score and 5 the highest. The total score presented is out of 40.

## RESULTS

Our search string resulted in 31 studies found in total across all databases postduplicate removal. Seven studies fulfilled our inclusion and exclusion criteria for the systematic review and data extraction.^11-17^ The Kappa score for interrater reliability was 1, indicating perfect inter-reviewer agreement. The review process is shown in the Figure. The mean (±SD) score for the critical appraisal using the CCAT was 70% (±14%). The score of each category and study is illustrated in Table 1.

**Table 1.** Critical Appraisal of the Included Studies Using the Crowe Critical Appraisal Tool v1.4

Study	Preliminaries	Introduction	Design	Sampling	Data collection	Ethical matters^a^	Results	Discussion	Total (%)
Chang et al^14^	3	4	4	4	5	0	5	1	26 (65%)
Das et al^15^	3	4	3	4	5	0	5	0	24 (60%)
Ravikumar et al^16^	3	3	3	4	5	0	5	0	23 (58%)
Gupta et al^11^	5	4	4	5	5	0	5	3	31 (78%)
Om et al^12^	5	5	5	5	5	0	5	5	35 (88%)
Song et al^13^	5	5	5	5	5	0	5	5	35 (88%)
Rivera et al^17^	3	2	4	4	5	0	4	1	23 (58%)

Each category is scored out of 5 points. ^a^As no ethical approval is needed for analysis of publicly available content, this section is absent from the studies and thus scores a zero.

Table 2 provides the characteristics of the studies. All the studies were published in 2021 and 2022, and the country of origin was the United States. The studies examined the following areas: gender-affirming surgery (*n* = 1), breast reconstruction using fat grafting (*n* = 1), aesthetic surgical procedures (abdominoplasty, blepharoplasty, breast augmentation, and rhinoplasty; *n* = 1), plastic surgeon profiles (*n* = 1), and profiles of videos relating to plastic surgery hashtags (*n* = 3).

**Table 2.** Study Characteristics

Author	Year	Country	Journal	Title	Field
Chang et al^14^	2022	USA	*Plastic and Reconstructive Surgery*	Analysis of TikTok's most viewed #PlasticSurgery content: an opportunity for educational outreach	General plastic surgery
Das et al^15^	2021	USA	*Plastic and Reconstructive Surgery*	Plastic surgeons in TikTok: top influencers, most recent posts, and user engagement	Plastic surgeons
Ravikumar et al^16^	2021	USA	*Plastic and Reconstructive Surgery*	Is TikTok the new Instagram? Analysis of plastic surgeons on social media	Plastic surgeons
Gupta et al^11^	2022	USA	*Breast/Trunk: Archives of Plastic Surgery*	A cross-sectional analysis of breast reconstruction with fat grafting content on TikTok	Breast reconstruction
Om et al^12^	2021	USA	*Aesthetic Surgery Journal*	Analyzing the quality of aesthetic surgery procedure videos on TikTok	Cosmetic procedures
Song et al^13^	2022	USA	*Plastic and Reconstructive Surgery—Global Open*	Evaluating the quality and reliability of gender-affirming surgery videos on YouTube and TikTok	Gender-affirming surgery
Rivera et al^17^	2022	USA	*Plastic and Reconstructive Surgery—Global Open*	Presence of cosmetic and aesthetic surgery on TikTok	Cosmetic and aesthetic surgery

Table 3 provides information with respect to the methodology used to collate the content, its characteristics, and quality appraisal where applicable. In total, 1105 videos were analyzed in the selected studies, of which 543 (49.1%) were created by physicians, and the other creators included nonphysicians, patients, companies, nurses, and clinics. Of the physician created videos: 410 (75.5%) were plastic surgeons, 91 (16.8%) did not report the specialty/other specialty, 36 (6.6%) were otolaryngologists, 5 (0.9%) were dermatologists, and 1 (0.2%) was a gynecologist.

**Table 3.** Content Analysis, Characteristics, and Methodology

Author	Methodology	No. of videos	Video statistics	Creator distribution	Physician specialty	Plastic surgery domain	Content type	Methodology for video assessment	Quality results
Chang et al^14^	Query #plasticsurgery and select top 200 videos based on engagement select	200	Likes: 190,000,000 Comments: 1,700,000	Physician: 109 (54.5%) General public: 37 (18.5%) Patients: 40 (20%) Nurse: 6 (3%) Company: 5 (2.5%) Other: 3 (1.5%)	Plastic surgeon: 109 (100%)	Cosmetic: 158 (79%) General plastic surgery: 20 (10%) Reconstructive: 18 (9%) Gender affirmation: 3 (1.5%) Burn: 1 (0.5%) Hand: 0 (0%)	Educational: 81 (40.5%) Patient: 38 (19%) Opinion: 33 (16.5%) Marketing: 19 (9.5%) Celebrity news: 19 (9.5%) Spam: 10 (5%)	None	NA
Das et al^15^	Top 10 plastic surgeons based on search	50	Followers: 23,756,800 Total no. of likes: 431,900,000 Top 5 video likes: 2,641,822 Comments: 47,501	Physician: 10 (100%)	Plastic surgeon: 10 (100%)	General plastic surgery: 50 (100%)	Educational: 38 (76%) Personal: 10 (20%) Advertising: 2 (4%)	None	NA
Ravikumar et al^16^	Query “plastic surgeon” and keep verified, US-based, board-certified plastic surgeons. Run the following hashtags: #plasticsurgery, #plasticsurgeon, #rhinoplasty, #liposuction, #cosmeticsurgery, #bodycontouring, #brazilianbuttlift, #abdominoplasty, #aestheticsurgery, #cosmeticsurgeon, #mastopexy, #buttaugmentation, #breastaugmentation, #botox, #fillers, #facelift, #tummytuck, #nosejob, #boobjob, #breastlift, #breastimplants, #aestheticsurgeon, #rhytidectomy	49	Mean followers: 47	Physician: 22 (12%)	Plastic surgeon: 22 (100%)	NA	Patient/surgery: 15 (30.6%) Self-promotional: 4 (8.2%) Educational: 14 (28.6%) Comedy: 16 (32.7%)	None	NA
Gupta et al^11^	Query “breast reconstruction with fat grafting” and retrieve top 200 videos	131	Likes: 1,871,980 Comments: 41,113 Shares: 58,662	Physician: 51 (39%) Nonphysician: 77 (59%) Company: 3 (2%)	No mention	NA	Personal: 77 (59%) Educational: 49 (37%) Advertising: 5 (4%)	DISCERN score	All videos: 2.16 Physician: 2.48 Nonphysician: 1.99 Company: 1.77 Personal experience: 2.03 Educational: 2.62 Advertising: 2.11
Om et al^12^	Query #rhinoplasty, #nosejob #blepharoplasty, #eyelidsurgery, #breastaugmentation, #breastimplant, #boobjob, #adbominoplasty, #tummytuck	200	Views: 4,800,000,000 Likes: 76,246,843	Physician: 112 (56%) Layperson: 88 (44%)	Plastic surgeon: 76 (68%) Otolaryngologist: 34 (30%) Other: 2 (2%)	Rhinoplasty: 50 (25%) Blepharoplasty: 50 (25%) Breast augmentation: 50 (25%) Abdominoplasty: 50 (25%)	Comedy: 24 (12%) Education: 86 (43%) Personal: 57 (28.5%) Before & After: 33 (16.5%)	DISCERN score	Rhinoplasty: 1.55 Blepharoplasty: 1.44 Breast Augmentation: 1.25 Abdominoplasty: 1.29
Song et al^13^	#(mastectomy or “top surgery”) AND #(GAS or transmales or transmen), #(breast augmentation) AND #(GAS or transfemales or transwomen), #(metoidioplasty) AND #(GAS or trans-females or transwomen), #(phalloplasty) AND #(GAS or transfemales or transwomen), and #(vaginoplasty) AND #(GAS or transfemales or transwomen)	55	Not recorded	Patient: 49 (89.1%) Physician: 6 (10.9%)	Plastic surgeon: 6 (100%)	Masculinizing top surgery: 36 (65.5%) Metoidioplasty: 15 (27.3%) Phalloplasty: 1 (1.8%) Vaginoplasty: 3 (5.5%)	Only aggregated view of TikTok + YouTube	DISCERN score	All videos: 2.14 Masculinizing top surgery: 2.1 Metoidioplasty: 2.2 Phalloplasty: 3 Vaginoplasty: 2
Rivera et al^17^	Query #plasticsurgery, #bbl, #nosejob, #rhinoplasty, #plasticsurgeon, #tummytuck, #liposuction, #facelift, #breastaugmentation, #cosmeticsurgery, #breastimplant, #abdominoplasty, #bodycontouring, #breastlift, #liquidrhinoplasty, #mastopexy, #necklift, #eyebrowlift, #aestheticsurgery, #cosmeticsurgeon, #aestheticsurgeon	420	Likes: 433,000,000 Comments: 3,870,000	Physician: 215 (51.2%) Nonphysician: 200 (47.6%) Private clinic: 5 (1.2%)	Plastic surgeon: 169 (78.6%) Nonboard certified plastic surgeon: 38 (17.6%) Dermatologist: 5 (2.3%) Otolaryngologist: 2 (0.9%) Gynecologist: 1 (0.5%)	Not mentioned	Promotional: 253 (60.2%) Educational: 105 (25%) Personal: 62 (14.8%)	None	NA

The most common types were educational (373, 34%), advertising/promotional/marketing (283, 26%), patient/personal experience (259, 23%), comedy (40, 4%), and other (150, 14%). “Other” included unclassified, opinion, celebrity news, spam, and before and after videos.

The videos were assessed in only 3 studies,^11-13^ and the DISCERN scale was used in all cases. The mean DISCERN score across all the videos (*n* = 386) analyzed was 1.91 (range: 1.08-3.00), which is classified as poor. Physician videos were found to have significantly higher scores than nonphysician videos across all the studies; however, their scores were still classified as average or poor. At the creator level, Om et al reported the lowest DISCERN score at 1.08 relating to breast augmentation videos created by a layperson, and the highest score at 1.81 to rhinoplasty videos created by plastic surgeons.^12^ Across the 3 studies, the highest score reported was 3 relating to a single phalloplasty video.^13^

## DISCUSSION

TikTok is a growing social media platform with millions of users daily, with the average TikTok user spending 90 min per day on the platform.^18^ Considering the short duration of video content, a user may be exposed to hundreds of videos per day. This systematic review assessed the current literature exploring TikTok's content within the plastic surgery domain.

Previous studies have demonstrated that patients are influenced by social media to undergo cosmetic and aesthetic procedures and that social media is routinely used prior to medical consultation.^19,20^ It is evident that plastic surgery videos perform very well within the TikTok community with high viewing numbers, and in combination with the platform's personalized algorithm (for the “For You Page”), a user might be exposed to numerous related videos based on their viewing history.^21^

Video content on TikTok ranged from educational to personal experience, and even self-promotional/advertising. It has previously been emphasized that it should be the plastic surgeon's responsibility to use the platform responsibly, rather than for a sole entertainment-related purpose.^22,23^ A recent literature review by Atiyeh et al concluded that plastic surgeons are reliant on social media for the advertisement and reach of their practice, and it is a steadily increasing trend; however, there is a high potential for misuse, leading to potential professional, legal, and ethical ramifications such as patient confidentiality being compromised.^24^

With respect to the quality of the content, only 3 out of the 7 selected studies undertook an assessment, and they all used the DISCERN instrument.^25^ Although the DISCERN tool is not typically used for social media videos, a lot of literature has used this instrument for the assessment of such videos.^26^ However, such a tool will not be of use in the regulation and monitoring of videos being uploaded.

The content of those videos may be improved to be more accurate and complete by attaching literature, references, and further links in the description of the videos, in combination with the creator painting a complete picture, for example, describing results, risks, and the surgery itself of a procedure. Dissemination of inaccurate medical information is a potential hazard common to all social media platforms and has previously been reported on TikTok during the COVID-19 pandemic with respect to the virus and the vaccines.^27^

The present study is limited by the primary studies’ reliance on appropriate titles and coding of content. The nature and ease of content sharing on social media platforms mean that some content may not have been captured by their search terms due to inappropriate labeling or sequential uploading as part of a playlist. Furthermore, the use of the DISCERN tool in the evaluation of quality educational content may not be the optimum means of analysis, and further work is needed to develop more versatile evaluation tools to keep up with innovations in content creation and sharing.

Recognition of the growing user-base and social amplification of content via TikTok is fundamental to understanding public perceptions of plastic surgery. As such, TikTok represents a potent tool for content sharing, which may be leveraged effectively to deliver important lifestyle advice, as well as sharing content between professionals. However, ensuring the accuracy of such information is paramount, and regulators may look to introduce content validation tools from recognized professionals to introduce a peer-review element within the platform.

**Figure.**
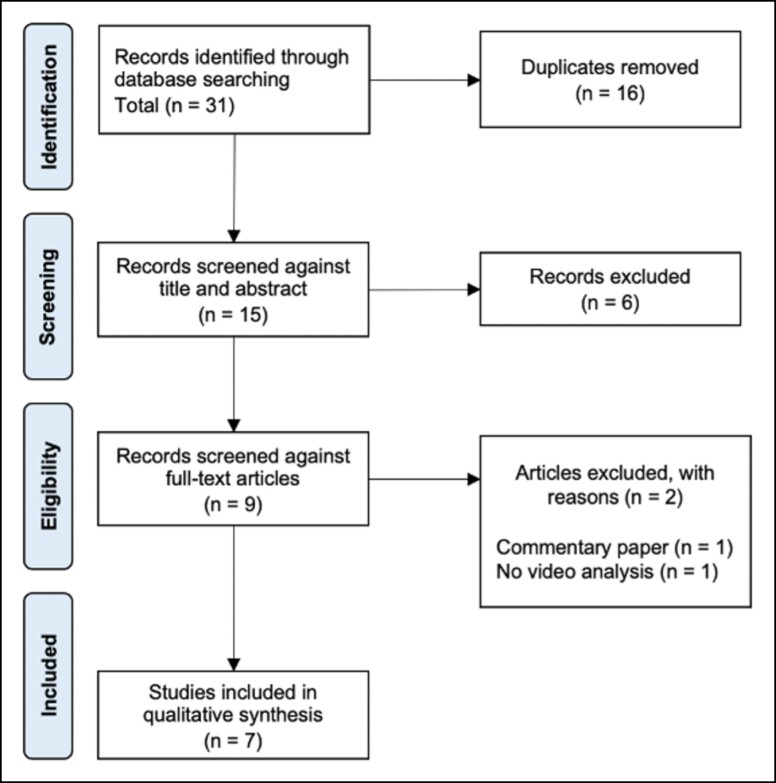
Flow chart of the reviewing process.

## CONCLUSIONS

TikTok is a growing platform in the domain of plastic surgery, both from a physician and a patient perspective. However, there is limited regulation of content, leading to potential issues with the accuracy of the information shared. The literature demonstrates low educational scores for appraised content, although there is a need for novel evaluation tools tailored to the type of content promoted on TikTok. Given its vast reach, TikTok has the potential to be utilized as a potent content sharing tool within plastic surgery both with the public and between professionals, although regulators may consider introducing peer-reviewing functions for healthcare content to prevent the spread of misinformation and uphold patient safety.
